# Determination of β-Galactooligosaccharides (GOS) in Infant Formula and Adult Nutritionals: Single-Laboratory Validation, First Action 2021.01

**DOI:** 10.1093/jaoacint/qsab095

**Published:** 2021-07-22

**Authors:** Denis Cuany, Fikrey Andetsion, Xavier Fontannaz, Thierry Bénet, Véronique Spichtig, Sean Austin

**Affiliations:** Société des Produits Nestlé S.A., Nestlé Research, Vers-Chez-Les-Blanc, 1000 Lausanne, Switzerland; Société des Produits Nestlé S.A., Nestlé Research, Vers-Chez-Les-Blanc, 1000 Lausanne, Switzerland; Société des Produits Nestlé S.A., Nestlé Research, Vers-Chez-Les-Blanc, 1000 Lausanne, Switzerland; Société des Produits Nestlé S.A., Nestlé Research, Vers-Chez-Les-Blanc, 1000 Lausanne, Switzerland; Société des Produits Nestlé S.A., Nestlé Research, Vers-Chez-Les-Blanc, 1000 Lausanne, Switzerland; Société des Produits Nestlé S.A., Nestlé Research, Vers-Chez-Les-Blanc, 1000 Lausanne, Switzerland

## Abstract

**Background:**

β-Galactooligosaccharides (GOS) are typically used in infant formula and adult nutritionals as a source of nondigestible oligosaccharides, which may bring beneficial effects through modulation of the gut microbiota. However, suitable methods for the determination of GOS in products with a high background of lactose do not exist.

**Objective:**

The aim of this work was to develop a method suitable for the determination of GOS in infant formula and adult nutritionals and demonstrate suitability through single laboratory validation.

**Methods:**

Reducing oligosaccharides are labeled with 2-aminobenzamide (2AB), separated by hydrophilic interaction LC, and determined assuming that all oligosaccharides give an equimolar response in the detector. The same sample is analyzed a second time after treatment with β-galactosidase to remove GOS. The difference in the determined oligosaccharides between the two measurements will be the GOS content of the sample. The method was validated in a single laboratory on infant formula and adult nutritionals.

**Results:**

Recoveries were in the range 91.5–102%, relative standards of deviation (RSD_r_) were in the range 0.7–5.99%, and one sample had an RSD_r_ of 8.30%. Except for the one sample with an RSD_r_ of 8.30%, the performance is within the requirements outlined in the *Standard Method Performance Requirements*, which specifies recoveries in the range 90–110% and RSD_r_ of below 6%.

**Conclusions:**

The method is suitable for the determination of GOS in infant formula and adult nutritionals.

**Highlights:**

A method has been developed which is suitable for the determination of GOS in products with a high background concentration of lactose (infant fromula and adult nutritionals). The method does not require access to the GOS ingredient used for the production of the finished product. It is also possible to separately quantify the amount of GOS containing three or more monomeric units in order to support dietary fibre analysis.

##  

When concentrated solutions of lactose are treated with β-galactosidase, it favors the production of β-galactooligosaccharides (GOS), since the enzyme transfers the galactose residue from lactose onto another carbohydrate acceptor instead of the usual water acceptor (1, 2). The resulting GOS are nondigestible, and in vitro and in vivo data indicate that the GOS may confer health benefits through the prebiotic effect (3–11). In particular, the GOS appear to be bifidogenic (4, 5, 12–15), and as such have been widely used in infant formula, to mimic some of the effects of human milk oligosaccharides (HMOs) (5, 13, 14, 16–18).

There are few methods available for the analysis of GOS in food products. In 2002 de Slegte (19) published a validated method. The method employs high-performance anion exchange chromatography with pulsed amperometric detection (HPEAC-PAD) to determine glucose, galactose, and lactose in a product. The product is then hydrolyzed using a β-galactosidase to transform all the GOS and lactose to glucose and galactose. The increase in galactose after hydrolysis is used to determine the GOS content after correction for the galactose released from lactose. This method has been evaluated in an inter-laboratory study and is the AOAC *Official Method*^SM^  **2001.02** (20). However, the method has a couple of weak points. Since the method is based on analyzing only the released galactose, the user needs to know the relative amounts of galactose to glucose in the GOS to correct for the non-analyzed glucose. Furthermore, because the method relies on measuring the difference in released galactose before and after hydrolysis, it is not well adapted to analyzing products containing high background concentrations of galactose or lactose, such as those found in infant formula. The method was improved by Hui et al. in 2018 (21), to encompass the analysis of both glucose and galactose and thus avoid the need for knowledge of the glucose to galactose ratio of the GOS ingredient. However, the basic principles of the method remain the same, and the updated method remains ill-suited to the analysis of GOS in samples with a high background of lactose.

In 2010 Albrecht et al. (22) demonstrated that GOS could be well separated and analyzed using capillary electrophoresis. The authors achieved promising results with products, including infant formula, but did not attempt method validation. In 2014 Austin et al. (23) developed a method for the analysis of GOS by hydrophilic interaction LC (HILIC). The method was based on the approach for the analysis of glycoprotein glycans described by Guile et al. (24). Oligosaccharides were extracted from the sample in water, and then labeled with 2-aminobenzamide (2AB). After labeling, maltodextrins were removed by treatment with amyloglucosidase; then the labeled oligosaccharides were separated by HILIC and the 2AB tag was detected using a fluorescence detector (FLD). Since it is the 2AB tag that is detected, and each oligosaccharide carries only a single tag, the detector signal is proportional to the molar concentration of the oligosaccharides. This enables the quantification of GOS without the need for analytical standards for every individual oligosaccharide present. The method performance was assessed through single-laboratory validation (SLV) and the results were promising. However, a major issue was the nonspecificity of the method, since the 2AB tag will label any reducing oligosaccharide with an aldose at the reducing end. The work described here is based on that method. To introduce specificity for GOS, two analyses are performed, one with and one without treatment with β-galactosidase. This once again has the disadvantage that we are measuring GOS by difference. However, since the method targets oligosaccharides, there is no significant overabundance of background that needs to be subtracted (as in the case of methods targeting galactose after hydrolytic release), so the results are more reliable.

## Experimental

### Validation Design

Table  1 summarizes the main requirements described in *Standard Method Performance Requirements* (SMPR^®^) 2014.003 (25) for the Determination of GOS in Infant Formula and Adult Nutritionals. The SLV was designed to test the method against those requirements. Reproducibility cannot be assessed in an SLV; however, intermediate reproducibility was assessed, and provided a guide as to whether the reproducibility targets might be achievable.

**Table 1.** *Standard Method Performance Requirements* (SMPR^®^) for the determination of GOS in Infant Formula and Adult Nutritionals^a^

Analytical range	0.2–3.0 g/100 g^b^
LOQ	≤0.2 g/100 g*b*
Repeatability (RSD_r_, %)	≤6
Reproducibility (RSD_R_, %)	≤12
Recovery, %	90–110

a From SMPR 2014.003 (25).

b Concentrations apply to the product as consumed (i.e., on reconstituted powders or concentrates or “as is” on ready to feed (RTF) products).

Materials from the SPIFAN II Single-Laboratory Validation Kit were used during the validation study and are listed in Table  2. The kit contains five products described as placebo products and 14 products described as fortified products. Four of the fortified products were found to contain GOS. It was also found that one of the placebo products contained GOS. Five additional matrices were added to increase the number of samples containing GOS. All samples were stored in the original packaging in a dry place, protected from light until the moment of use. For the spike–recovery experiments three GOS ingredients were donated by FrieslandCampina, Clasado, and Nestlé. The GOS content of these ingredients was established by analysis according to Method **2001.02** (19, 20), and results were as follows:

$$\begin{array}{c} \text{GOS}\;1\;\left(\text{GOS}\;\text{content}:\;45.1\;g/100\;g,\;\text{RSD}:\;4.742\%\right).\\ \text{GOS}\;2\;\left(\text{GOS}\;\text{content}:\;46.8\;g/100\;g,\;\text{RSD}:\;3.105\%\right).\\ \text{GOS}\;3\;\left(\text{GOS}\;\text{content}:\;51.8\;g/100\;g,\;\text{RSD}:\;2.368\%\right). \end{array}$$

Prior to analysis, all powder products were reconstituted by dissolving 25 g powder in 200 g water and all ready-to-feed (RTF) products were used as is. The in-house reference sample was analyzed without prior reconstitution.

**Table 2.** Samples used during the single-laboratory validation

No.	Product description	Sample type
Placebo products		
1	Child formula powder	Powder
2	Infant element powder	Powder
3	Adult nutritional RTF^a^, high-protein	Liquid
4	Adult nutritional RTF, high-fat	Liquid
5	Infant formula RTF, milk-based	Liquid
Fortified products		
6	SRM 1849a	Powder
7	Infant formula powder, partially hydrolyzed milk-based	Powder
8	Infant formula powder, partially hydrolyzed soy-based	Powder
9	Toddler formula powder, milk-based	Powder
10	Infant formula powder, milk-based	Powder
11	Adult nutritional powder, low-fat	Powder
12	Child formula powder	Powder
13	Infant elemental powder	Powder
14	Infant formula powder, FOS^b^/GOS-based	Powder
15	Infant formula powder, milk-based	Powder
16	Infant formula powder, soy-based	Powder
17	Infant formula RTF, milk-based	Liquid
18	Adult nutritional RTF, high-protein	Liquid
19	Adult nutritional RTF, high-fat	Liquid
Additional products		
20	Infant formula powder with GOS	Powder
21	Infant formula powder with GOS	Powder
22	Infant formula powder with GOS/FOS	Powder
23	RTF adult nutritional with GOS	Liquid
24	Infant formula powder with partially hydrolyzed protein	Powder
25	Infant formula powder with GOS/HMO (laboratory reference)	Powder

a RTF = Ready to feed.

b FOS = Fructooligosaccharides.

#### (a)

 *Calibration fit*This was assessed by injecting calibration solutions of maltotriose (11–2100 nmol/mL) at 11 different concentrations, prepared in triplicate, and all containing the laminaritriose internal standard. The ratio of the peak areas (maltotriose/laminaritriose) was plotted against the concentration of maltotriose (since the laminaritriose concentration remained constant throughout there was no need to plot the ratio of the concentrations on the *x*-axis). A linear model was used to fit the data for calibration purposes. The differences between actual concentration and the concentration predicted by the calibration model were calculated and plotted against the analyte concentration.

#### (b)

 *Recovery*This was assessed on three GOS-free matrices from the SPIFAN kit (samples No. 12, 13, and 19) and one additional GOS-free product (sample No. 24) that covered both infant formula and adult nutritionals (Table  3). Fructooligosaccharides (FOS) were present in two samples (No. 12 and 19). The samples were analyzed without spiking (level 0) and spiked with three different GOS materials (GOS 1 or GOS 2 or GOS 3), at four spike levels. Four HMOs (2’-fucosyllactose (2’FL), lacto-N-neotetraose (LNnT), 3’-sialyllactose (3’SL), and 6’-sialyllactose (6’SL)) were also added to assess potential interferences (Table  3).The samples were analyzed in duplicate on three different days by two different operators on two different instruments.

**Table 3.** Design of spike–recovery experiment

Sample description				Amount of GOS or HMO spiked				
GOS spike level	Sample No.	HMO spike	GOS Type	GOS, g/100 g	2’FL, mg/100 g	LNnT, mg/100 g	3’SL, mg/100 g	6’SL, mg/100 g
0	19	N	No GOS	0.000	0.0	0.0	0.0	0.0
0	24	N	No GOS	0.000	0.0	0.0	0.0	0.0
0	12	N	No GOS	0.000	0.0	0.0	0.0	0.0
0	13	N	No GOS	0.000	0.0	0.0	0.0	0.0
0	19	Y	No GOS	0.000	20.6	8.5	6.1	11.5
0	24	Y	No GOS	0.000	109.7	54.3	25.1	69.0
0	12	Y	No GOS	0.000	219.4	106.3	65.3	140.8
0	13	Y	No GOS	0.000	21.9	8.6	6.0	13.3
1	19	N	GOS 3	0.226	0.0	0.0	0.0	0.0
1	24	Y	GOS 1	0.205	21.9	8.6	6.0	13.3
1	12	Y	GOS 2	0.203	109.3	54.1	25.0	68.8
1	13	Y	GOS 3	0.231	205.5	106.2	65.3	140.6
2	19	Y	GOS 2	0.586	205.5	110.1	65.9	139.3
2	24	Y	GOS 3	0.655	110.8	54.8	25.4	69.7
2	12	Y	GOS 1	0.571	21.9	8.6	6.0	13.3
2	13	N	GOS 2	0.570	0.0	0.0	0.0	0.0
3	19	Y	GOS 1	0.913	107.0	53.0	27.3	72.2
3	24	Y	GOS 2	1.044	219.3	108.5	63.3	140.7
3	12	Y	GOS 3	1.152	21.9	8.6	6.3	13.3
3	13	N	GOS 1	1.015	0.0	0.0	0.0	0.0
4	19	N	GOS 2	3.01	0.0	0.0	0.0	0.0
4	24	N	GOS 3	3.00	0.0	0.0	0.0	0.0
4	12	N	GOS 1	3.01	0.0	0.0	0.0	0.0

#### (c)

 Repeatability (r) and intermediate reproducibility (iR)These were assessed by analyzing samples (containing GOS) in duplicate on at least six different days by two different operators on two different instruments. The in-house statistical package Q-Stat was used to calculate the SD_r_ and SD_iR_ using Equations 1 and 2:

(1) $$SD_{r}=\sqrt{\frac{\sum_{i=1}^{n}SD_{i}^{2}}{n}\;}=\sqrt{\frac{\sum_{i=1}^{n}{\left(x_{i1}-x_{i2}\right)}^{2}}{2n}}$$

 

(2) $$\;SD_{iR}=\sqrt{SD^{2}\left(b\right)+\frac{1}{2}\times SD^{2}(r)}$$

 where: *n* = number of (single or duplicate) determinations; *x_i_* = individual result within the set of single determinations with i going from 1 to *n*; *x_i1_* ,*x_i2_* = two results within the set of duplicate determination with i going from 1 to *n*; *SD(b)* = SD between the means of duplicates; *SD_r_* = SD for repeatability; and *SD_iR_* = SD for intermediate reproducibility.

#### (d)

 LOD and LOQBecause the method requires the analysis of a complete profile of oligosaccharides, the detection and quantification limits depend on the GOS profile as well as the concentration of GOS in the product, making it extremely difficult to assess (except on an individual oligosaccharide basis). To demonstrate method applicability at the LOQ defined in the SMPR (25), spike–recovery experiments have been performed at the desired LOQ (0.2 g/100 g).

In addition to the spike–recovery experiments, LOD and LOQ were assessed based on the measurement of blank samples. In the SPIFAN kit 14 samples were included that did not contain GOS. Each of these was analyzed in duplicate to generate a mean and SD for each blank sample. The SDs of the blank results were combined according to Equation 1. The LOD and LOQ were then estimated using Equations 3 and 4:
 

(3) $$\mathrm{LOD}=GOS_{\mathrm{blk}}+SD_{\mathrm{blk}}\times 3$$

 

(4) $$\mathrm{LOQ}=GOS_{\mathrm{blk}}+SD_{\mathrm{blk}}\times 10$$

where *GOS_blk_* = largest GOS content measured in the 14 blank samples and *SD_blk_* = combined SD of all the blank samples.

In addition, the LOQ for a single oligosaccharide was assessed using the maltotriose standard by measuring the S/N when low concentrations of maltotriose were injected on the system, the S/N versus concentration was plotted, and the LOQ assigned at the concentration where S/N was 10. This was assessed on two different instruments.

**AOAC *Official Method*** ^**SM**^  **2021.01**

β-Galactooligosaccharides in Infant Formula and Adult Nutritionals

Liquid Chromatographic Method

First Action 2021

[Applicable to the determination of 0.2–3.0 g/100 g of β-galactooligosaccharides (GOS) in reconstituted or ready-to-feed (RTF) infant formula or adult nutritionals. May underestimate GOS concentration if there is a significant amount of nonreducing GOS present.]

*See*  Tables **2021.01A** and **2012.01B** for the results of the single-laboratory validation (SLV) supporting acceptance of the method for first action.

*Caution:* The method employs corrosive, toxic (acute and irritant), and flammable chemicals such as acetic acid, sodium hydroxide, formic acid, ammonia solution, and acetonitrile. Amyloglucosidase is a health hazard and ammonia solution is also dangerous for the environment. Sodium cyanoborohydride (NaBH_3_CN) is very toxic, highly flammable, and dangerous for the environment. Wear personal protective equipment such as gloves and safety glasses. Perform all manipulations under a fume hood. Refer to the materials safety data sheets, take appropriate additional safety precautions for handling, and ensure waste disposal is in accordance with local requirements.

### A. Principle

Samples are reconstituted in water and oligosaccharides present in samples are extracted at 70°C. Duplicate aliquots of the diluted sample are taken and both are treated with amyloglucosidase to hydrolyze any maltooligosaccharides present (Assay 1). One aliquot is also treated with β-galactosidase (Assay 2) to hydrolyze all the GOS present. An internal standard (laminaritriose) is added to both aliquots and the oligosaccharides are fluorescently labeled with 2-aminobenzamide (2AB). Labeled extracts are diluted with acetonitrile prior to injection on an ultra high perfromance liquid chromatography (UHPLC) system equipped with a fluorescence detector (FLD) and a hydrophilic interaction LC (HILIC) analytical column. The analytes are separated using a gradient of aqueous ammonium formate in acetonitrile and detected with an FLD. An external maltotriose calibration curve is prepared in the same way as the samples but without enzymatic treatment. Since it is the 2AB label that is detected, each oligosaccharide has an equivalent molar response. The maltotriose calibration curve can thus be used to determine the molar concentrations of the oligosaccharides in the two assays. It is then necessary to know the MW of each signal in the chromatogram to convert the molar concentrations to mass concentrations. This can be done by coupling a mass spectrometer (but once a GOS ingredient profile has been characterized by HILIC-FLD-MS, future samples can be analyzed without the MS). They may also be estimated by comparing the relative retention time (RRT) of the oligosaccharide against that of a dextran ladder, similar to the approach used for glycan analysis (24). The GOS content is obtained by subtracting the oligosaccharide (OS) content obtained in Assay 2 from the OS content obtained in Assay 1.

### B. Apparatus

- *Analytical balance*.—Weighing to ±0.1 mg (Mettler-Toledo, Greifensee, Switzerland).
- *Weighing boats*.
- *Volumetric flasks*.—10 to 1000 mL.
- *Glass tubes*.—10 or 20 mL.
- *pH* *meter*.—Reading 0.1 pH (Metrohm, Herisau, Switzerland).
- *Microcentrifuge tubes*.—1.5 mL, safe lock or screw cap (Eppendorf, Hamburg, Germany).
- *Microcentrifuge tubes*.—2 mL, safe lock or screw cap (Eppendorf).
- *Floating rack*.—For microtubes (Nalgene, Thermo Fisher Scientific, Waltham, MA, USA).
- *Water bath*.—At 70 ± 1°C (Thermo Fisher Scientific).
- *Water bath*.—At 65 ± 1°C (Thermo Fisher Scientific).
- *Water bath*.—At 60 ± 1°C (Thermo Fisher Scientific).
- *Centrifuge*.—For 1.5 and 2 mL microtubes able to operate at 10 000 × *g* (Eppendorf).
- *Micropipettes with tips*.—0.02 to 10 mL (Socorex Isba, Ecublens, Switzerland).
- *Vortex mixer*.—Scientific Industries (Bohemia, NY, USA).
- *Ultrasonic bath*.
- *UHPLC column*.—Acquity UPLC BEH Glycan, 1.7 µm; 2.1 mm × 150 mm (Waters, Milford, MA, USA).
- *Liquid chromatography instrument*.—Equipped with a gradient pump able to deliver a flow of 0.3 to 0.6 mL/min with a back-pressure up to 15 000 psi, an online degasser, an autosampler equipped with a refrigerated sample compartment, a temperature-controlled column compartment able to maintain a stable temperature of 25.0 ± 1.0°C, and an FLD (Thermo Fisher Scientific).

### C. Chemicals and Reagents

- *Deionized water*.—18 MΩ Milli-Q (Merck-Millipore) purified or equivalent.
- *Maltotriose (with accurately known purity).—*e.g., Ultrapure (Carbosynth, Newbury, UK). In case of issues, check the moisture content and purity following the procedure described in Annex A.
- *Laminaritriose*.—>90% (Me gazyme, Bray, Ireland).
- *Glacial acetic acid anhydrous*.—GR for analysis (Merck-Millipore).
- *Sodium hydroxide pellets*.—Merck-Millipore.
- *Acetonitrile*.—Gradient grade for LC (Merck-Millipore).
- *Dimethylsulfoxide* *(DMSO)*.—Puriss p.a. (Sigma-Aldrich, St Louis, MO, USA).
- *Anthranilic acid amide (2-aminobenzamide, 2AB)*.—Purum (Sigma-Aldrich).
- *Sodium cyanoborohydride*.—Purum (Sigma-Aldrich).
- *Amyloglucosidase* (*Aspergillus niger*).—9 U/mg (Roche Diagnostics, Rotkreuz, Switzerland: 11 202 367 001).
- *β-Galactosidase* (*Aspergillus niger*).—4000 U/mL (Megazyme E-BGLAN).
- *Formic acid*.—GR for analysis (Merck-Millipore).
- *Ammonium hydroxide solution 25–30%*.—GR for analysis (Merck-Millipore).
- *Dextran*.—With average MW 1000 Da (Fluka, Darmstadt, Germany).
- *Isomaltose*.—Carbosynth, Compton, UK

### D. Preparation of Reagents

- *Maltotriose (malto-3) stock solution (about 10* *µmol/mL).—*Weigh 100 mg malto-3 into a weighing boat and record the mass to 0.1 mg. Transfer quantitatively into a 20 mL volumetric flask with water and dilute to the volume with the same solvent.
- *Laminaritriose internal standard working solution (about 2* *µmol/mL).—*Weigh the whole content of a 50 mg laminaritriose vial into a weighing boat and record the mass to 0.1 mg. Transfer quantitatively into a 50 mL volumetric flask and complete to the mark with water.
- *Sodium hydroxide (1 M)—*Dissolve 10 ± 0.2 g sodium hydroxide pellets in 200 mL water in a 250 mL volumetric flask. After cooling to room temperature, make up to the mark with demineralized water and mix well.
- *Sodium acetate buffer (0.2 M, pH 4.5).—*Into a large beaker (>500 mL) containing 400 mL demineralized water, pipette 5.8 mL glacial acetic acid. Adjust to pH 4.5 with sodium hydroxide solution 1 M. Transfer the solution to a 500 mL volumetric flask and make up to the mark with water.
- *Water—acetonitrile solution (25 + 75).—*Add 50 ±1 mL water to 150 ±1 mL acetonitrile in a glass bottle and mix.
- *2AB labeling reagent.—2AB* *(0.35 mol/L)–NaBH_3_CN* *(1.0 mol/L)* *in DMSO—acetic acid* *(70 + 30)* *solution.—*Pipette the volume of DMSO and glacial acetic acid into a 20 mL glass tube according to the number of tests to perform (*see*  Table **2021.01C** for quantities). Mix the solution using a vortex mixer. Weigh the amount of 2-aminobenzamide (2AB) and sodium cyanoborohydride (NaBH_3_CN) (*see*  Table **2021.01C**) in a 10 mL glass tube, and then add the corresponding volume of 30% acetic acid in DMSO. Mix (vortex) and use an ultrasonic bath for complete dissolution (about 10 min).
- *Amyloglucosidase solution (60 U/mL in 0.2 M sodium acetate buffer pH 4.5).—*Weigh an amount of amyloglucosidase corresponding to 600 ± 20 U and dissolve with 10.0 mL sodium acetate buffer 0.2 M, pH 4.5. This solution is prepared on the day of use and kept at 4°C until use.
*Note*: For the development and validation of this method, amyloglucosidase No. 11202367001 available from Roche Diagnostics, was used. Enzyme activities may vary slightly from one batch to the other (units/mg are mentioned on the label). Adapt the weight of enzyme in order to reach a concentration of 60 ± 6 U/mL. Another amyloglucosidase, No. 10102857001, also available from Roche Diagnostics, has also been tested and found to be suitable. This enzyme is already in suspension (140 U/mL) and can be diluted with 0.2 M sodium acetate buffer pH 4.5 in order to produce a working concentration (60 U/mL). When enzymes from another source are used it is imperative to ensure the enzymes employed will completely hydrolyze any maltodextrins in the product without hydrolyzing any analytes, as well as not showing any interference in the chromatogram. This can be checked by performing an analysis with maltodextrin as a sample, a GOS ingredient as a sample, and running a blank with the amyloglucosidase only.
- *β-Galactosidase solution (4000 U/mL).—*Use the solution as is.
*Note:* For the development and validation of this method, the β-galactosidase E-BGLAN, available from Megazyme, was used. When enzyme from another source is used it is imperative to ensure the enzyme employed will completely hydrolyze the GOS without hydrolyzing any other oligosaccharides that may be present in the sample.
- *Dextran solution.—*Weigh about 20 mg isomaltose and about 50 mg dextran 1000 into a weighing boat. Transfer into a 50 mL volumetric flask with water and dilute to the volume with the same solvent.

### E. Mobile Phase Preparation

- *Eluent A.—*Acetonitrile.
- *Eluent B.—Ammonium formate (100 mM, pH 4.4).—*Add 4.6 ± 0.1 g (3.78 mL) formic acid (100%) in a beaker containing 800 mL water. Adjust the pH to 4.40 ± 0.05 with ammonium hydroxide solution (25–30%). Transfer quantitatively to a 1000 mL volumetric flask and dilute to the volume with water.

### F. Preparation of Standards

Prepare a six-level calibration curve by diluting the maltotriose stock solution as described in Table **2021.01D**, using volumetric flasks made up to the final volume with water.

### G. Sample Preparation

- For analysis of products on an RTF basis reconstitute powder or liquid concentrates according to instructions. For example, weigh 25 g infant formula powder into a bottle and add water to a final total weight of 225 g. Place the mixture in a water bath at 70°C for 25 min under constant stirring. Cool the solution to room temperature.
- For reconstituted products (as prepared above), or products that are sold as RTF, weigh an amount of sample (m) containing a maximum of 40 mg GOS, but not more than 5 g of sample, into a 25 mL (V) volumetric flask and make up to the mark with water.
- For analysis of homogeneous powder products without prior reconstitution, weigh an amount of sample (m) containing a maximum of 80 mg GOS, but not more than 1.1 g of powder, into a 50 mL (V) volumetric flask. Add water (30 mL) and heat at 70°C with constant agitation for 25 min. Cool to room temperature and complete to the mark with water.
- *Standard calibration curve.—*With each series of analyses, prepare a maltotriose calibration curve (six-level, Table **2021.01D**). Into a microtube (1.5 mL), transfer 500 µL standard solution. Add 250 µL water. Mix (vortex) and place in a water bath at 60°C for 2 h. At the end of the incubation time, mix (vortex), place at 4°C for 5–10 min and then continue with the standards from step **G(h)**.
- *Dextran ladder.—*With each series of analyses, prepare a dextran ladder. Into a microtube (1.5 mL), transfer 500 µL dextran solution. Add 250 µL water, mix (vortex) and then continue with the dextran ladder from step **G(h)**.
- *Hydrolysis of maltodextrins (Assay 1 and Assay 2) and GOS (Assay 2).—*Into two microtubes (1.5 mL) marked A1 and A2, transfer 500 µL sample solution. Add 200 µL amyloglucosidase solution (60 U/mL in 0.2 M sodium acetate buffer pH 4.5) to both tubes. Add 50 µL water to tube A1 and 50 µL β-galactosidase solution to tube A2. Mix (vortex) and place in a water bath at 60°C for 2 h ± 5 min. At the end of the incubation time, put all the tubes with β-galactosidase (Assay 2) in a boiling water bath for 5–6 min to stop the reaction. Then mix (vortex) and place at 4°C for 5–10 min.
- *Reagent blank.—*With each series of analyses, prepare a reagent blank by performing the whole procedure on water instead of the sample solution (Assay 1 and Assay 2).
- *Internal standard addition.—*Centrifuge all tubes (standard cuvre, Assay 1, Assay 2, blank 1, blank 2, and dextran ladder) for 10–20 s at 10 000 × *g* to remove drops from the lid. Add 100 µL internal standard laminaritriose (2.0 µmol/mL) to all the tubes and mix well (vortex).
- *Derivatization.—*Transfer 20 µL of solutions containing internal standard into 2 mL microtubes (safe lock or screw cap) and add 200 µL 2AB labeling reagent to each tube. Mix (vortex) and place the tubes in a water bath at 65 ± 1°C for 2 h ± 5 min. After 2 h mix the tubes (vortex) and then place at 4°C for 5–10 min.
- *Dilution.—*Once they are cooled, centrifuge for 10–20 s at 10 000 × *g* to remove drops from the lid. Carefully open the microtubes under a fume hood (because of possible release of HCN) and dilute by addition of 1 mL acetonitrile–water (75 + 25) solution. Mix well (vortex) and then centrifuge for 5 min at 10 000 × *g* before transferring 1 mL supernatant to an injection vial.

### H. Chromatographic Conditions

The UHPLC system is equipped with an Acquity UPLC BEH Glycan column (2.1 mm × 150 mm, 1.7 µm). The column is held at 25 ± 1°C and the injection volume is 2 µL. The analytes are separated using the gradient described in Table **2021.01E** and are detected by means of an FLD tuned at the following wavelengths: excitation λ = 330 nm and emission λ = 420 nm.

### I. System Suitability Check

Before starting an analysis allow the chromatographic system to equilibrate and the FLD to warm up (if necessary) under the initial conditions, for at least 15 min. Let the derivatized standard and sample solutions equilibrate to the autosampler temperature before making any injections. Ensure the system pressure and baseline are stable and there are no leaks. Before starting a series of analysis, make at least one injection of the dextran ladder, and check the RRTs of the oligosaccharides. The RRTs should be in the following range: isomaltose 0.56–0.60, isomaltotriose 1.50–1.57, isomaltotetraose 1.92–2.12, isomaltopentaose 2.16–2.40, and isomaltohexaose 2.29–2.58.

### J. Calibration and Calculations

It is recommended to use bracketed calibration, injecting three standards followed by a maximum of 10 samples, then three standards, etc. For example, inject standards at levels 1, 3 and 5, then 10 samples, then standards at levels 2, 4 and 6, then 10 samples, then standards 1, 3, 5, etc.

Use the instrument software to plot a six-point standard curve of “instrument response for maltotriose/instrument response for laminaritriose” against “concentration of maltotriose” in the standard (in µmol/mL). Fit a linear model to the data including the origin as a point (but not forced through the origin).

When integrating the peaks in the chromatogram it is important to make an estimate of the S/N of the smaller peaks. Only include peaks with an S/N of 10 or greater in the calculations. Smaller peaks cannot be quantified accurately and introduce inaccuracies in the measurement if included. Do not integrate the lactose peak or the maltose peak (if present). In Assay 2, a peak may appear in the region that was covered by the lactose signal in Assay 1; do not integrate this peak.

Use the standard curve to calculate the molar concentration (in µmol/mL) of each oligosaccharide in the chromatogram (C_m_) without β-galactosidase treatment (Assay 1) and calculate the total oligosaccharides in that sample using Equation 5. Then use the standard curve to calculate the molar concentration (in µmol/mL) of each oligosaccharide in the chromatogram (C_m_) after β-galactosidase treatment (Assay 2) and calculate the total oligosaccharides in that sample according to Equation 6. Then calculate the GOS content of the sample using Equation 7. If it is desired to calculate the dietary fiber content of the GOS, exclude the disaccharides from the calculations in Equations 5 and 6:

(5) $$C_{\mathrm{TOS}}=\sum (C_{m}\times MW)\times \frac{V}{m}\times 0.0001$$

 

(6) $$C_{B}=\sum (C_{m}\times MW)\times \frac{V}{m}\times 0.0001$$

 

(7) $$C_{\mathrm{GOS}}=C_{\mathrm{TOS}}-C_{B}$$

where: C_TOS_ = total concentration of oligosaccharides in the untreated sample (in g/100 g); C_B_ = total concentration of oligosaccharides in the enzyme-treated sample (in g/100 g); C_GOS_ = total concentration of GOS in the sample (in g/100 g); C_m_ = molar concentration of each individual oligosaccharide in the sample (in µmol/mL); MW = MW of each individual oligosaccharide in the sample, either estimated from the glucose unit (GU) value (Annex B) or measured by MS (Annex C); V = volume to which the original sample weight was diluted (in mL); m = weight of sample diluted to volume (V) (in g); and 0.0001 = factor to convert the result from µg/g to g/100 g.

### K. Annex A—Assessment of Maltotriose Concentration

In general, the manufacturer’s certificate of analysis (CoA) provides sufficient information to accurately calculate the concentration of the maltotriose stock solution (having corrected for both the moisture content and the purity of the standard). However, in some cases the CoA may not be available, or the data provided may be inaccurate. In these cases, the purity and moisture content of the maltotriose standard needs to be assessed.

- *Moisture determination.—*The moisture content is assessed by Karl Fischer titration using a system adapted to measuring small quantities of water.
- *Additional apparatus.—*
  (1)  Karl Fischer apparatus for measuring small amounts of water (e.g., Metrohm 89 9 Coulometer, Metrohm, Herisau, Switzerland).
  (2) Glass tubes (10 mL) with rubber stoppers for air-tight sealing.
  (3)  Gas-tight syringe (1 mL).
- *Additional chemicals.—*
  (1) *Hydranal* *formamide*.—Honeywell Research Chemicals (Charlotte, NC, USA) or equivalent.
  (2) *Hydranal* *Coulomat AD*.—Honeywell Research Chemicals, or equivalent.
- *Procedure.—*
  (1)  Prepare a solvent mixture of Hydranal formamide and Hydranal Coulomat AD (1 + 1 by volume) sufficient for the number of analyses to be carried out (∼5 g/analysis + 2 g).
  (2)  Accurately weigh the maltotriose sample (200 ± 20 mg) into a glass tube, and seal it with the rubber stopper.
  (3)  Add 5 g solvent mixture and dissolve the sample (use a vortex mixer and/or sonic bath).
  (4)  Weigh 500 mg solvent mixture (without sample) in a syringe and inject it into the coulometer to determine the moisture content of the solvent.
  (5) Repeat the analysis of the blank solvent mixture two more times, to have a total of three measurements.
  (6)  Weigh 500 mg sample solution in a syringe and inject into the coulometer to determine the moisture content of the sample + solvent.
  (7)  Repeat the analysis of the sample solution.
  (8) Calculate the moisture content of the sample by subtracting the average moisture content of the solvent mixture from the average moisture content of the sample + solvent.
- *Purity determination.—*The purity of the maltotriose is assessed by analyzing the standard prepared at level 6 (expected concentration approximately 1400 nmol/mL). The standard is prepared and injected on the chromatographic system as described in the main method. The chromatogram is then assessed for signals in addition to the ones expected (maltotriose and the internal standard, laminaritriose). The peak areas are measured for all peaks except for laminaritriose and assigned a corresponding molecular mass depending on the identity of the signal [180 for glucose, 342 for disaccharides composed of two hexoses (Hex 2), 504 for Hex 3 (including maltotriose), 666 for Hex 4, 828 for Hex 5, 990 for Hex 6, 1152 for Hex 7; *see*  Figure **2021.01A**]. The peak area of maltotriose multiplied by its molecular mass (504) is then divided by the sum of all the peak areas multiplied by their corresponding masses to calculate the purity of maltotriose. This is best illustrated by an example.

**Figure 2021.01A.**
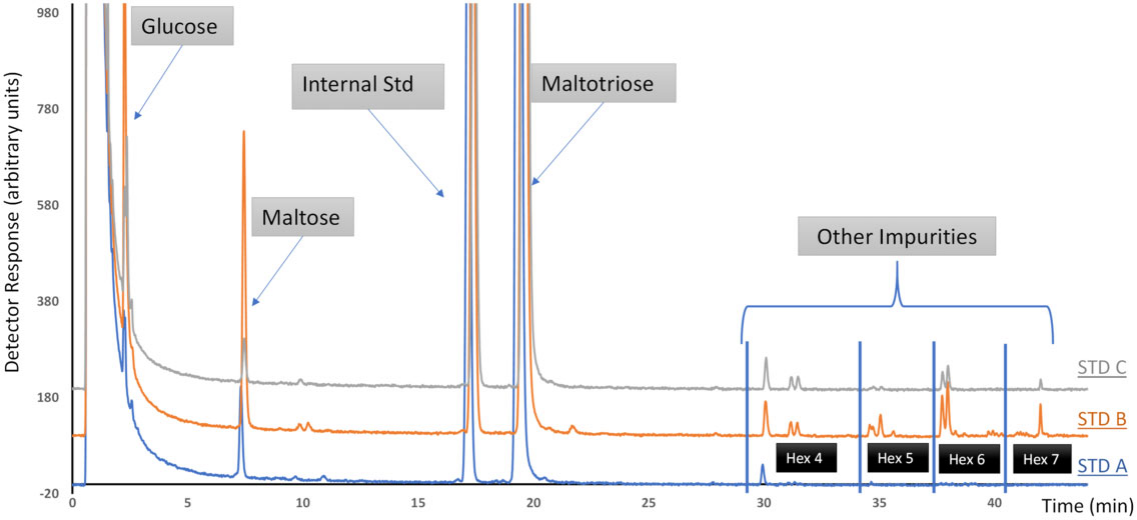
Example chromatograms from the assessment of the purity of maltotriose standards. Hex = Hexose. Std, STD = Standard.

An example data set is shown in Table **2021.01F**; taking these data the purity of the maltotriose would be calculated as shown in Equation 8.

(8) $$P_{M3}(\%)=\frac{A_{M3}\times MW_{M3}}{\sum_{i=1}^{n}A_{Mi}\times MW_{Mi}}\times 100=\frac{40320}{43362}\times 100=93.0\%$$

where: *P_M3_(%)* = purity of maltotriose in %; *A_M3_* = peak area of maltotriose; *MW_M3_* = MW of maltotriose in g/mol; *A_Mi_* = peak area of signal with i hexose units; and *MW_Mi_* = MW of oligosaccharide with i hexose units in g/mol.

If the measured moisture and purity are in good agreement with the manufacturer’s CoA (each ±2 g/100 g), it is recommended to use the data provided on the manufacturer’s CoA. If the difference is larger it is recommended to use the measured moisture and purity or to use a different batch of maltotriose.

### L. Annex B—Determination of Molecular Weight Using the Dextran Ladder

To determine the MW of each signal it is possible to calibrate the column using the dextran ladder. The dextran oligosaccharides elute from the smallest to the largest. Isomaltose is the first signal and is composed of 2 GU, and it is therefore assigned a GU value of 2; the next oligosaccharide of the dextran ladder has a GU of 3, and so on (Figure **2021.01B**).

**Figure 2021.01B.**
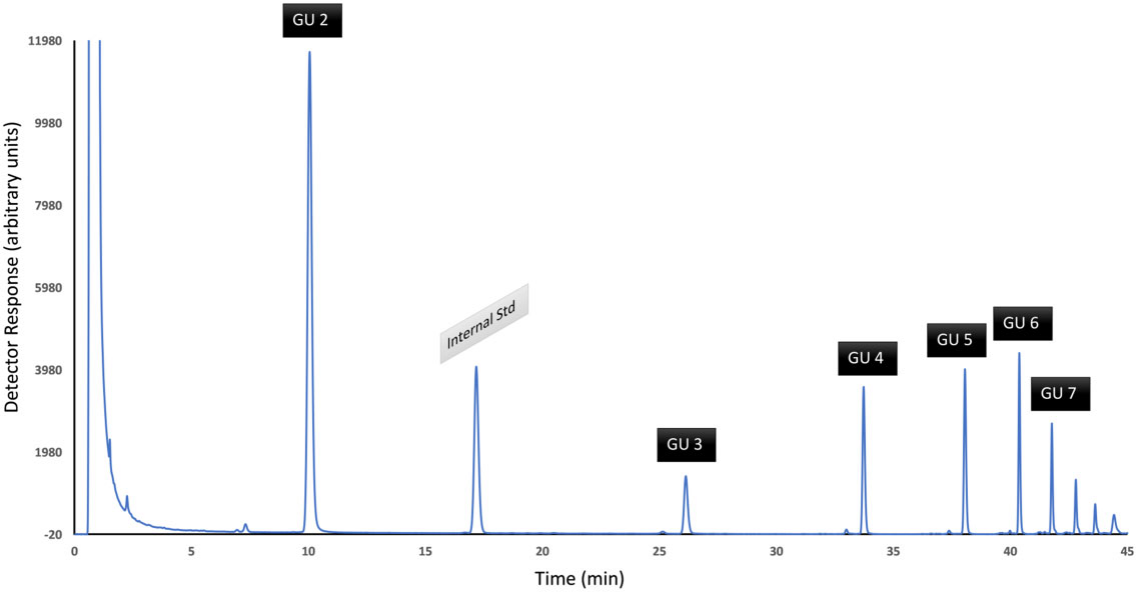
Typical chromatogram of the dextran ladder with the glucose unit (GU) assigned to each peak. Std = Standard.

Determine the RRT of each of the dextran signals compared to the internal standard according to Equation 9. Then make a plot of GU against RRT and fit a third-order polynomial (*y* = a*x*^3^ + b*x*^2^ + c*x* + d). For each signal in the sample chromatogram calculate the RRT in the same way as for the dextran ladder, and then assign a GU value from the polynomial. The molecular mass of each signal can then be assigned based on the GU value using Table **2021.01G**.

(9) $$\mathrm{RRT}(Dn)=\frac{RT(Dn)}{RT(IS)}$$

where: *RRT(Dn)* = RRT of the signal n in the dextran ladder; *RT(Dn)* = retention time of signal n in the dextran ladder; and *RT(IS)* = retention time of the laminaritriose internal standard.

### M. Annex C—Determination of MW Using LC-MS

- Protocol for MW determination of GOS by UHPLC–FLD–MS.—
  1. *Sample preparation.—*The same vial prepared for the quantitative determination of GOS can also be used for the analysis of MW assignment. Alternatively, one may prepare a sample using only the GOS ingredient. In case the mass spectrometer has insufficient sensitivity, it is possible to prepare a sample having 10 times greater GOS concentration for the purposes of peak identification only (in this case it is recommended to use the GOS ingredient).
  2. *Mass spectrometer setup.—*In addition to the UHPLC–FLD instrument, a mass spectrometer is required. Use the same chromatography setup and conditions as described in the main method but split the flow eluting from the analytical column in a ratio of about 1:1. One half of the flow is passed through the FLD, and the other half is directed to a mass spectrometer. (*Note:* if you connect the mass spectrometer in series after the FLD there is a high chance that the flow cell will rupture.)
    The following describes the setup of the API 4000 QTrap mass spectrometer (AB Sciex, Framingham, MA, USA) in our laboratory. The mass spectrometer settings in other laboratories should be optimized locally.
  3. *LC parameters.—*Use the same LC conditions as described in the quantitative method.
    The injection volume can be increased up to 10 µL.
  4. *MS parameters.—Experiment type*.—Multiple ion monitoring, monitoring the masses listed in Table **2021.01H**.

Mode: ESI negative.

Curtain Gas (GUR): 17.0

Ion Spray Voltage (IS): −3800 V

Ion Source Gas 1 (GS1): 60.0

Ion Source Gas 2 (GS2): 20.0

Ion Source Heater Temperature (IHT): 400.0°C

Declustering Potential (DP): −60

Entrance Potential (EP): −10.0 

In some cases, there may be some overlap of peaks having a different MW. In these cases, the analyst should assign the MW that would be expected to result in the least error (e.g., if the MS signals of a trisaccharide and tetrasaccharide overlap, but the signal is stronger for the trisaccharide, then assign the peak the MW of a trisaccharide).

## Results

### Method Development

We already developed and validated a method for the analysis of GOS in infant formula (23). The method is based on the labeling of the oligosaccharides with a fluorescent tag, separating the labeled oligosaccharides by HILIC and then detecting the label. Assuming each labeled oligosaccharide has an equimolar response factor, the GOS content could be calculated using a surrogate oligosaccharide standard (maltotriose). The disadvantage with the approach is the lack of specificity of the method since the tag will label any oligosaccharide with an aldose at the reducing end. To overcome this issue the sample was split in two aliquots and treated with enzymes. The first enzyme, amyloglucosidase, is added to both aliquots, and removes maltodextrins, thus reducing the background oligosaccharide concentration. The second enzyme, β-galactosidase, is added only to the second aliquot and removes the GOS. The purity of the enzymes is extremely important. The amyloglucosidase should not have any activities that hydrolyze GOS, and the β-galactosidase needs to be specific for GOS. We found that amyloglucosidases commonly used for dietary fiber analysis were well suited for the selective and specific removal of maltodextrins. However, some of the enzymes introduced additional signals in blank chromatograms, which we wanted to avoid. We found that the amyloglucosidase enzymes from Roche worked well for this application. The β-galactosidases were less specific, so when selecting an enzyme, screening for side activities is recommended. We found the β-galactosidase from Megazyme (E-BGLAN, 4000 U/mL) was suitable for the application.

When processing the chromatogram, all peaks need to be individually integrated, and it is important to exclude any peaks that have an S/N ratio below 10. Lactose is generally present in very large quantities resulting in a large signal in Assay 1. The lactose signal should not be integrated. After β-galactosidase treatment (Assay 2), the lactose peak is mostly removed, but a peak may remain in the window where lactose had been in Assay 1. This peak should not be integrated in Assay 2 because if present in Assay 1 it would be under the lactose and thus not quantified.

During the method validation, we used different batches of maltotriose from different suppliers to prepare the calibration curve. Since we had established a reference sample during development, we observed results biases that were linked to the batch of maltotriose used for calibration. We first suspected that the standards may have absorbed moisture during storage, so the moisture content of the standards was checked using a Karl Fischer titration and compared against the manufacturer’s CoA. In general, we found the measured moisture contents of the standards were in good agreement with the manufacturer’s CoA. We therefore decided to check the purity of the samples. We suspected that the most likely contaminants in maltotriose would be other maltooligosaccharides; this is easily investigated using the method developed for the GOS analysis. So, the maltotriose standards were labeled with 2AB and the oligosaccharide separated using the same chromatographic system as used for the GOS. We observed that most of the maltotriose standards contained maltose and glucose, and some signals that eluted later (Figure **2021.01A**). The concentration of the signals was calculated, assuming equimolar response factors, and it was found that for some batches of maltotriose standard, the measured purity was different from the purity stated on the CoA (Table  4). The standards delivering expected results on the reference sample were the standards for which the measured maltotriose purity was in good agreement with the CoA. It is therefore recommended that the purity of the maltotriose calibration standard be checked in a similar manner before use. When the purity measurement is within ±2 g/100 g of the CoA it is recommended to use the value stated on the CoA; if the difference is greater, it is recommended to use the measured purity, or to use a different batch of maltotriose.

**Table 2021.01A.** Summary of results from the precision study

No.	Sample description	*n*	Mean concn, g/100 g	RSD_r_, %	RSD_iR_, %	Target RSD_r_, %	Meets target (Y/N)
5	Infant formula RTF, milk-based	6 × 2	0.216	2.09	5.81	≤6	Y
7	Infant formula powder, partially hydrolyzed milk-based	6 × 2	0.357	0.72	5.06	≤6	Y
14	Infant formula powder, FOS/GOS-based	6 × 2	0.333	3.36	6.67	≤6	Y
15	Infant formula powder, milk-based	6 × 2	0.300	5.99	7.36	≤6	Y
17	Infant formula RTF, milk-based	6 × 2	0.211	1.89	6.37	≤6	Y
20	Infant formula powder with GOS	6 × 2	0.436	3.11	4.94	≤6	Y
21	Infant formula powder with GOS	6 × 2	0.277	1.33	7.81	≤6	Y
22	Infant formula powder with GOS/FOS	6 × 2	0.769	2.50	4.90	≤6	Y
23	Adult nutritional RTF with GOS	6 × 2	0.664	8.30	9.80	≤6	N
25	Infant formula powder with GOS/HMO (lab reference sample)	12 × 2	6.53^a^	1.30	2.70	≤6	Y

All results reported on a “ready-to-feed” basis except.

a concentration in the nonreconstituted powder.

### Method Specificity

Specificity of the method is achieved by running the analysis before and after hydrolysis with a β-galactosidase and making the difference between the two analyses. Potentially interfering oligosaccharides are not hydrolyzed by the β-galactosidase, and thus are not considered as part of the GOS.

GOS is frequently found in products along with FOS. The FOS are either nonreducing oligosaccharides, and thus cannot be labeled, or they contain a reducing-end fructose, which is a ketose, and is not labeled with 2AB under the labeling conditions used. The FOS are thus not detected either before or after β-galactosidase treatment. Two of the samples used for spike–recovery experiments contained FOS and there were no interferences. Polydextrose is another oligosaccharide that may be found in GOS-containing products. When polydextrose was analyzed alone, or when spiked (at 0.4 g/100 g) into a GOS-containing sample, no interfering signals were observed. Polydextrose does not have a well-defined structure so we are not sure why this oligosaccharide is not observed. We postulate that it either has a molecular mass range beyond that of the GOS, or that there are no reducing ends susceptible to being labeled.

**Figure 1.**
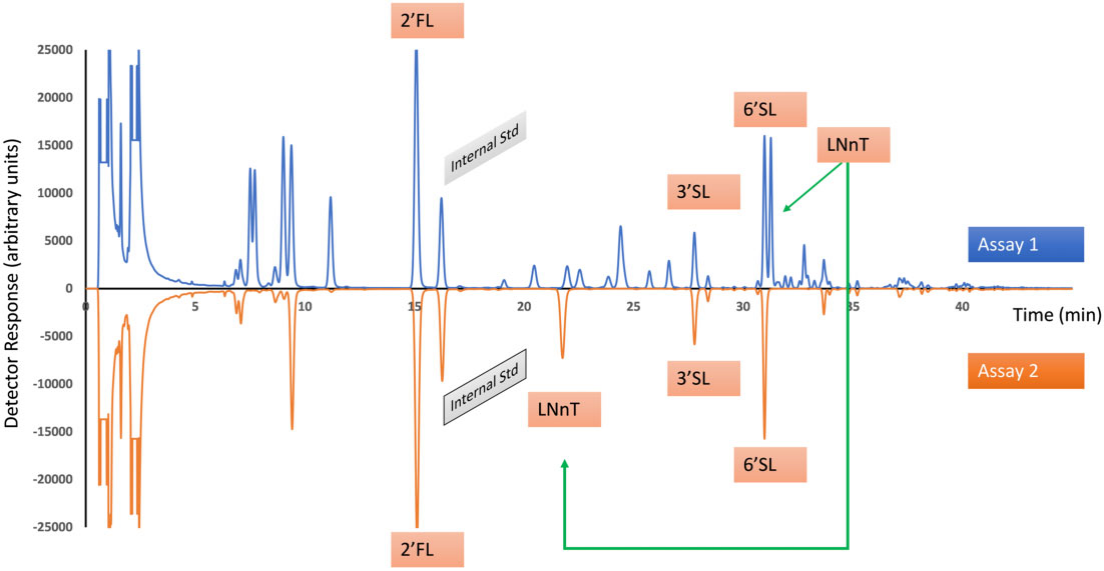
Mixture of GOS and HMO before (Assay 1) and after (Assay 2) β-galactosidase treatment. Std = Standard.

HMOs are emerging ingredients likely to be found in infant formula, potentially in combination with GOS. They give clear signals in the chromatogram and are not removed by treatment with β-galactosidase; thus they do not interfere with the analysis. However, HMO containing terminal galactose residues (e.g., lacto-N-tetraose (LNT) LNnT, lacto-N-hexaose (LNH), etc.) can be partially hydrolyzed, since the terminal galactose on the nonreducing end of the oligosaccharide can be removed by the β-galactosidase. This means that the oligosaccharide appears in one place in the chromatogram in Assay 1 and in a different place in Assay 2, meaning that the MW assignment will change between the two assays, and thus the GOS may be slightly overestimated. LNT and LNnT typically run in the region with the tetrasaccharides, so would be assigned as a Hex 4 in Assay 1 (Figure  1). In Assay 2 they can move to the Hex 3 region (Figure  1). It is thus important to identify the hydrolysis product in Assay 2 and assign the peak the mass of Hex 4 to correct for this shift. If using a mass spectrometer to identify the signals, this is readily done, since both the tetrasaccharide and the trisaccharide contain an *N*-acetylhexosamine (HexNAc) residue, which can be clearly differentiated from the oligosaccharides containing only hexose (Hex) residues. In case of doubt, the analyst can include a sample of the pure HMO and identify the retention time before and after β-galactosidase treatment.

### Calibration Fit

**Figure 2.**
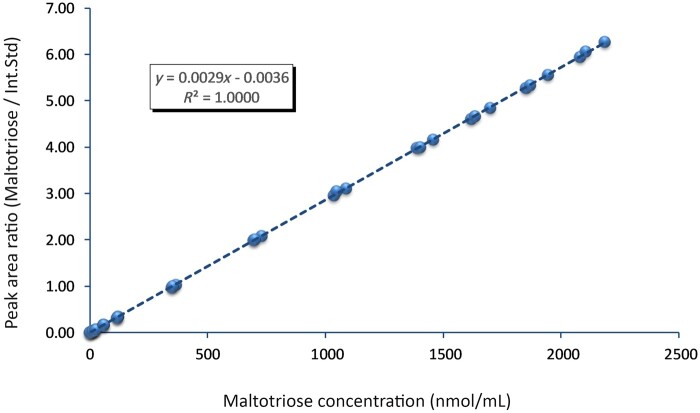
Maltotriose calibration curve Int. Std = Internal standard.

**Figure 3.**
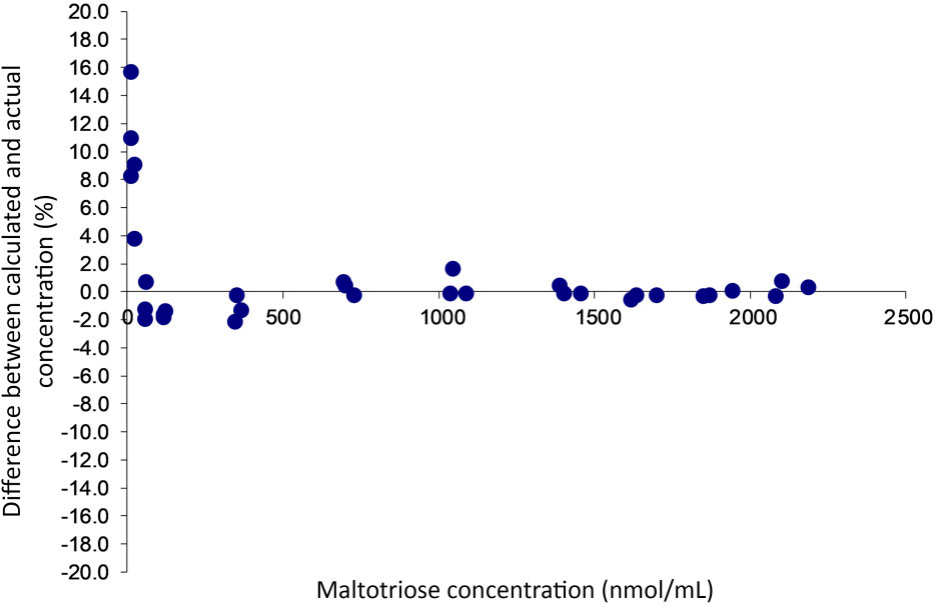
Plot of differences between predicted and actual concentrations for the calibration curve. Differences are generally below 5% except at 11 nmol/ml.

A linear model was used to fit the data for calibration purposes (Figure  2) and the model seems to fit the data well. The relative difference between the predicted and actual concentrations of the standards were calculated and plotted against the analyte concentration (Figure  3); in general the predicted concentration is within 5% of the actual concentration although there are a few outliers, particularly at lower concentrations. We therefore confirm that a linear calibration model is appropriate.

### LOD and LOQ

The LOD and LQQ are difficult to estimate since they depend on the profile of the GOS ingredient being used; therefore spike–recovery experiments were performed at 0.2 g/100 g, the required LOQ assigned in the SMPR (25). Spike–recovery results and the method precision at the addition level of 0.2 g/100 g were in line with the requirements of the SMPR, and thus it appears the method meets the LOQ requirements of the SMPR.

To try to estimate the LOD and LOQ, the GOS-free samples from the SPIFAN kit (14 samples) were reconstituted and analyzed in duplicate on a single day. The average measured GOS content of the 14 GOS-free samples was 0.00458 g/100 g (when considering negative results as 0). The highest GOS content measured in a blank was 0.0266 g/100 g. The combined SD for the blank results was 0.00596 g/100 g. Using the highest measured blank and the combined SD, the LOD and LOQ were estimated as:

$$\begin{array}{c} \text{LOD}=0.0266+3\times 0.00596=0.0444\;g/100\;g\\ \text{LOQ}=0.0266+10\times 0.00596=0.0861\;g/100\;g \end{array}$$

We also assessed the LOD and LOQ for a single oligosaccharide on two different instruments. Low concentrations of maltotriose solution (expected to be close to the LOQ) were prepared in the same way as the standards and injected on the two instruments. The S/N was measured and plotted against concentration. The concentration of maltotriose giving rise to a peak with an S/N of 10 was defined as the LOQ. On one instrument the concentration of maltotriose resulting in an S/N of 10 was 9 nmol/mL, while on the other it was 3 nmol/mL.

The combined results demonstrate that the method LOQ is lower than 0.2 g/100 g and could be close to 0.1 g/100 g. The practical LOQ will depend on the GOS profile, and at which concentration it is no longer possible to accurately quantify the smaller signals in the profile. With three different ingredients we have demonstrated that the method can accurately determine GOS at a concentration of 0.2 g/100 g, and therefore meets the LOQ requirement of the SMPR.

### Precision

In order to establish which samples contained GOS, all samples from the SPIFAN kit were analyzed in duplicate on a single day, and five of the 19 samples were found to contain GOS, being:

No. 5, Infant formula RTF, milk-based.
No. 7, Infant formula powder, partially hydrolyzed milk-based.
No. 14, Infant formula powder, FOS/GOS-based.
No. 15, Infant formula powder, milk-based.
No. 17, Infant formula RTF, milk-based.
Five additional GOS-containing samples were added to the kit to increase the number of matrices in the precision study:
No. 20, Infant formula powder with GOS.
No. 21, Infant formula powder with GOS.
No. 22, Infant formula powder with GOS/FOS.
No. 23, Adult nutritional RTF with GOS.
No. 25, Infant formula powder with GOS/HMO.

The repeatability and intermediate reproducibility were assessed by analyzing 9 of the 10 samples after reconstitution in duplicate on six different days, using two different instruments (same model) and four different columns (the same stationary phase and manufacturer, but different batch) by two different analysts. The 10th sample (sample No. 25) was analyzed without reconstitution in duplicate on 12 different days (Table **2021.01A**).

The SMPR (25) requires that the RSD_r_ should be less than or equal to 6%. The achieved RSD_r_ was less than 6%, for all matrices except one (No. 23, Adult nutritional RTF), which had an RSD_r_ of 8.3%. The RSD_iR_ was less than 10% in all cases, suggesting that the method may be able to achieve an RSD_R_ of less than 12% during multilaboratory testing (as required in the SMPR).

### Accuracy/Trueness

Four different blank matrices were spiked at four levels with three different GOS ingredients (as described in Table  3). Recoveries were in the range 91.5–102% across the four matrices (Table **2021.01B**) and the three GOS ingredients (Table  5); thus the recoveries meet the requirements set out in the SMPR. When organized by GOS ingredient (Table  5), it appears that there may be a small impact on the recovery, with GOS 2 having slightly higher recoveries than the other two GOS ingredients. Since two of the spiked samples contain FOS, and HMOs were spiked into most samples (Table  3, Table **2021.01B**), these results demonstrate that the method works well in the presence of other oligosaccharides that may be present in the sample together with the GOS.

**Table 2021.01.B.** GOS recoveries in different matrices

No.	Sample description	Type of GOS	*n*	Spike, g/100 g	Measured, g/100 g	Recovery, %	RSD, % (Rec.)
19	Adult nutritional RTF, high-fat (contains FOS)	NA^a^	6	0	−0.01	NA	NA
		GOS 3	6	0.226	0.213	94.1	5.3
		GOS 2	6	0.586	0.583	99.4	3.7
		GOS 1	6	0.913	0.836	91.5	4.9
		GOS 2	6	3.01	2.90	96.2	3.3
24	Infant formula powder with partially hydrolyzed protein	NA	6	0	0.001	NA	NA
		GOS 1	6	0.205	0.196	95.4	5.6
		GOS 3	6	0.655	0.639	97.6	2.8
		GOS 2	6	1.04	1.02	98.0	3.7
		GOS 3	6	3.00	2.79	93.2	3.4
12	Child formula powder (contains FOS)	NA	6	0	0.001	NA	NA
		GOS 2	6	0.203	0.208	102	3.5
		GOS 1	6	0.571	0.535	93.7	5.3
		GOS 3	6	1.15	1.09	94.6	2.8
		GOS 1	6	3.01	2.94	97.9	5.3
13	Infant elemental powder	NA	6	0	0.002	NA	NA
		GOS 3	6	0.231	0.228	98.5	4.1
		GOS 2	6	0.570	0.569	99.8	3.5
		GOS 1	6	1.01	0.959	94.5	4.9

a NA = Not applicable.

### Check-Standards

During all series of analyses check-standards (three levels) were also analyzed (Table  6). The check-standards were prepared with different stock solutions of maltotriose from the ones used to prepare the calibration curve. In most cases the results of the check-standards were within ±5% of the expected concentration. On 2 days there were problems with the lowest check-standard; the difference from the expected value is 6.5% and 12.5%, but the results from the other check-standards and the reference sample on those 2 days were within expectations.

**Table 2021.01C.** Examples of quantities for 2AB reagent preparation

Max. number of tests	30% Acetic acid in DMSO		0.35 M 2AB—1 M NaBH_3_CN in 30% acetic acid in DMSO		
	DMSO, mL	100% Acetic acid, mL	30% Acetic acid in DMSO, mL	2AB, mg	NaBH_3_CN, mg
23	4.20	1.80	5.00	236 ± 5	314 ± 5
35	6.30	2.70	7.50	354 ± 10	471 ± 10
47	7.70	3.30	10.00	472± 10	628± 10
71	11.20	4.80	15.00	708 ± 10	942 ± 10
95	15.40	6.60	20.00	944± 20	1256± 20

### Impact of Approach for Peak Identification

The method requires that the molecular mass of each peak is determined, and two approaches for this are proposed. One approach is the use of a mass spectrometer, and this approach is the one that was used for the generation of the data described so far. This approach is the one most adaptable to different GOS ingredients, and once a profile has been established for a particular ingredient the specified peak masses can be applied without the need of a mass spectrometer for every analysis (since the peak pattern will remain constant). However, if a mass spectrometer is not available, it is also possible to calibrate the system using a dextran ladder. This approach is less flexible than the MS approach since the dextran ladder is used to define regions of the chromatogram where it is expected GOS having particular masses will elute. However, different GOS ingredients contain different oligosaccharides and the boundaries between where the different masses elute move between ingredients. The method is thus a compromise, with the boundaries between tri- and tetra-saccharides, between tetra- and penta-saccharides, etc., being set in a region that is not perfect for any single ingredient, but that returns acceptable data for a range of different ingredients. We compared the results using the two different approaches for peak assignment on the set of samples used for the precision study (Table  7). The data indicate that there is excellent agreement in results using the two different approaches. However, we noticed that all samples appeared to contain the same GOS profile (that of GOS 1) and the samples covered a limited concentration range. Therefore, we did the same exercise using some of the spiked samples (Table  8) and again the data show good alignment between the two approaches for peak assignment, at the three different spike levels and for the three different GOS ingredients.

**Table 2021.01D.** Dilution scheme for the preparation of the calibration curve and approximate concentration in the calibration curve

Standard	Volume of maltotriose stock solution, µL	Final volume, mL	Maltotriose concn^a^, nmol/mL
Level 1	80	20	35
Level 2	200	10	175
Level 3	400	10	350
Level 4	800	10	700
Level 5	1200	10	1150
Level 6	1600	10	1400

a This is the target concentration; calculate the actual concentration based on the actual concentration of stock solution prepared in the laboratory and adjust for the purity and moisture content of the standard being used.

**Table 2021.01E.** Chromatographic gradient

Time, min	Flow, mL/min	A (acetonitrile), %	B (ammonium formate 100 mM, pH 4.4), %
0.0	0.6	88.0	12.0
7.0	0.6	88.0	12.0
17.0	0.6	85.0	15.0
21.0	0.6	85.0	15.0
36.0	0.6	72.6	27.4
44.0	0.6	54.0	46.0
44.1	0.3	54.0	46.0
44.5	0.3	30.0	70.0
49.5	0.3	30.0	70.0
52.0	0.3	88.0	12.0
54.0	0.6	88.0	12.0
60.0	0.6	88.0	12.0

### Performance of AOAC Official Method^SM^ 2001.02

During the method validation, the samples used for spike–recovery were also analyzed using AOAC *Official* *Method* **2001.02** (19, 20) (Table  9). As expected, AOAC *Official* *Method* **2001.02** does not meet the requirements of the SMPR. In particular it struggles when the GOS concentrations are below 1 g/100 g, returning poor recoveries and poor precision. When concentrations are above 1 g/100 g, the spike–recovery results are generally good, but the precision of the method remains poor. The data confirm that the method is not well suited for the analysis of samples containing a high background of lactose and relatively low amounts of GOS. The new method should be recommended for analyzing such samples.

**Table 2021.01F.** Example data set for illustration of the calculation of maltotriose purity

Identity	MW, g/mol	Peak area	Peak area × MW
Glucose	180	2	360
Maltose	342	3	1026
Maltotriose	504	80	40 320
Hex 4	666	1	666
Hex 5	990	1	990
Sum	—	—	43 362

### Determination of the GOS Contribution to Dietary Fiber

Codex has proposed two definitions of dietary fiber, one which only includes nondigestible polysaccharides having a degree of polymerization (DP) of 10 or more, and one which includes nondigestible polysaccharides having a DP of 3 or more (26). GOS typically have DPs below 10, and thus they would not be considered dietary fiber in countries adopting that definition. However, many countries have adopted the definition including nondigestible polysaccharides with a DP ≥ 3. In this case a proportion of the GOS would meet the definition of fiber, but the nondigestible disaccharide components of GOS remain outside the fiber definition. For labeling the fiber contents of a product containing GOS, it is thus useful to be able to measure the GOS having a DP of 3 and above. With this method it is possible to do so by simply excluding the disaccharides from Equations 5 and 6. There is no existing reference method for this analysis, so we were only able to assess the precision of this measurement (Table  10). For the measurement of GOS having a DP ≥ 3, the RSD_r_ were quite comparable with those for GOS, being between 0.78 and 8.80% (for GOS 0.72–8.30%); however, the intermediate reproducibilities (RSD_iR_) were a bit higher, being 6.00–13.00% (for GOS 4.90–9.80%).

**Table 2021.01G.** Assignment of peak MW according to its GU value

GU range	GOS type	MW, g/mol
1.6–2.5	Hex 2	342
2.5–2.6	Internal standard	
2.6–3.4	Hex 3	504
3.4–4.2	Hex 4	666
4.2–5.2	Hex 5	828
5.2–6.2	Hex 6	990
>6.2	Hex 7	1152

**Table 2021.01H.** Masses monitored for the assignment of GOS masses

Q1 mass, Da	Dwell time, ms	Corresponding GOS	GOS MW, g/mol
461.3	50.0	Hex 2 (including lactose)	342
623.4	50.0	Hex 3	504
785.4	50.0	Hex 4	666
947.4	50.0	Hex 5	828
1109.5	50.0	Hex 6	990
1271.6	50.0	Hex 7	1152
1433.6	50.0	Hex 8	1314

**Table 4.** Comparison of maltotriose purity assessed by manufacturer and this method

Maltrotriose standard	CoA purity, %	CoA moisture, %	Measured purity, %	Measured moisture, %
Supplier A Batch 1	98	4.6	98.7	3.9
Supplier A Batch 2	98	3.2	90.2	4.4
Supplier A Batch 3	98.2	0.6	89.9	5.8
Supplier B Product 1	91	4.4	90.3	5.4
Supplier B Product 2	97	3.0	90.2	3.2
Supplier C Batch 1	95	2.1	96.6	2.5

**Table 5.** GOS recoveries by type of GOS ingredient

Type of GOS	No.	Matrix	*n*	Spike, g/100 g	Measured, g/100 g	Recovery, %	RSD, % (Rec.)
GOS 1	24	Infant formula powder with partially hydrolyzed protein	6	0.205	0.196	95.4	5.6
	12	Child formula powder	6	0.571	0.535	93.7	5.3
	19	Adult nutritional RTF, high-fat	6	0.913	0.836	91.5	4.9
	13	Infant elemental powder	6	1.01	0.959	94.5	4.9
	12	Child formula powder	6	3.01	2.94	97.9	5.3
GOS 2	12	Child formula powder	6	0.203	0.208	102	3.5
	13	Infant elemental powder	6	0.570	0.569	99.8	3.5
	19	Adult nutritional RTF, high-fat	6	0.586	0.583	99.4	3.7
	24	Infant formula powder with partially hydrolyzed protein	6	1.04	1.02	98.0	3.7
	19	Adult nutritional RTF, high-fat	6	3.01	2.90	96.2	3.3
GOS 3	19	Adult nutritional RTF, high-fat	6	0.226	0.213	94.1	5.3
	13	Infant elemental powder	6	0.231	0.228	98.5	4.1
	24	Infant formula powder with partially hydrolyzed protein	6	0.655	0.639	97.6	2.8
	12	Child formula powder	6	1.15	1.09	94.6	2.8
	24	Infant formula powder with partially hydrolyzed protein	6	3.00	2.79	93.2	3.4

## Conclusions

The method described is suitable for the determination of GOS in most infant formula and adult nutritional products within the concentrations typically used in such products. The precision (RSD_r_) and recoveries of the method generally meet the SMPR (25). Other oligosaccharides that may be found in infant formula such as maltodextrins, FOS, polydextrose and HMO do not interfere with the analysis. The method may also be applied for assessing the proportion of the GOS that meets the definition of dietary fiber.

**Table 6.** Results of check*-*standards

Day	Analyst	Column	Instrument	Δ of check-standard		
				Level 1 ∼80 nmol/mL, %	Level 2 ∼490 nmol/mL, %	Level 3 ∼980 nmol/mL, %
1	B	1663832618648	RS3	−1.2	−2.6	−1.5
2	B	16638308607	RS4	3.5	0.9	0.5
2	A	1663832618648	RS3	−1.0	−0.9	−1.5
3	B	16638308607	RS4	0.3	−2.9	1.8
4	A	1663832618648	RS3	−12.5	2.7	−0.8
5	B	16638308607	RS4	2.1	1.6	2.3
6	B	16638308607	RS4	1.2	2.4	1.7
7	B	16638308607	RS4	4.3	−2.8	−0.8
7	A	1663832618648	RS3	3.8	−2.7	−1.2
8	A	16638308607	RS4	1.2	−1.4	0.5
9	B	1663832618666	RS3	0.5	−2.9	−1.2
9	A	16638308607	RS4	−2.0	−1.6	−1.6
10	A	16638308607	RS4	−4.2	−3.2	−1.6
11	B	1663832618666	RS3	−2.0	−1.6	−1.6
12	A	16638308607	RS4	0.5	−2.6	−0.4
13	B	1663832618666	RS3	−0.7	−2.9	0.1
14	A	1663832618666	RS3	6.5	1.0	0.3
15	B	1683935218555	RS4	−3.4	−1.1	−0.9
16	A	1663832618666	RS3	1.4	2.5	3.0
17	A	1663832618666	RS3	0.3	−1.2	1.7

**Table 7.** Comparison of precision results with MW assigned by MS or dextran ladder^a^

No.	Sample description	MS spectrum			Dextran ladder		
		Mean concn, g/100 g	RSD_r_, %	RSD_iR_, %	Mean concn, g/100 g	RSD_r_, %	RSD_iR_, %
5	Infant formula RTF, milk-based	0.216	2.09	5.81	0.216	1.96	5.84
7	Infant formula powder, partially hydrolyzed milk-based	0.357	0.72	5.06	0.356	0.70	4.93
14	Infant formula powder, FOS/GOS-based	0.333	3.36	6.67	0.332	3.35	6.57
15	Infant formula powder, milk-based	0.300	5.99	7.36	0.299	6.01	7.33
17	Infant formula powder RTF, milk-based	0.211	1.89	6.37	0.211	2.01	6.52
20	Infant formula powder with GOS	0.436	3.11	4.94	0.435	3.07	4.83
21	Infant formula powder with GOS	0.277	1.33	7.81	0.276	1.43	7.95
22	Infant formula powder with GOS/FOS	0.769	2.50	4.90	0.768	2.50	4.80
23	Adult nutritional RTF with GOS	0.664	8.30	9.80	0.664	8.30	9.70

a All results reported on a “ready-to-feed” basis.

**Table 8.** Comparison of trueness results with MW assigned by MS or dextran ladder^a^

No.	Sample description	Type of GOS	Spike, g/100 g	MS spectrum			Dextran ladder		
				Measured, g/100 g	Recovery, %	RSD, % (Rec.)	Measured, g/100 g	Recovery, %	RSD, % (Rec.)
13	Infant elemental powder	GOS 1	1.01	0.959	94.5	4.9	0.958	94.4	5.1
12	Child formula powder (contains FOS)	GOS 1	3.01	2.94	97.9	5.3	2.96	98.3	5.2
12	Child formula powder (contains FOS)	GOS 2	0.203	0.208	102	3.5	0.202	99.6	3.3
19	Adult nutritional RTF, high-fat (contains FOS)	GOS 2	0.586	0.583	99.4	3.7	0.575	98.1	3.5
24	Infant formula powder with partially hydrolyzed protein	GOS 2	1.04	1.02	98.0	3.7	1.02	98.0	3.7
19	Adult nutritional RTF, high-fat (contains FOS)	GOS 2	3.01	2.90	96.2	3.3	2.89	95.9	3.3
13	Infant elemental powder	GOS 3	0.231	0.228	98.5	4.1	0.227	98.4	4.1
24	Infant formula powder with partially hydrolyzed protein	GOS 3	0.655	0.639	97.6	2.8	0.640	97.6	2.8
12	Child formula powder (contains FOS)	GOS 3	1.15	1.09	94.6	2.8	1.09	94.3	2.6
24	Infant formula powder with partially hydrolyzed protein	GOS 3	3.00	2.79	93.2	3.4	2.79	93.1	3.4

a All results reported on a “ready-to-feed” basis.

**Table 9.** Analysis of spike–recovery samples using AOAC *Official Method* **2001.02**

No.	Sample description	Type of GOS	*n*	Spike, g/100 g	Recovery, %	RSD_r_	RSD_iR_	Meets target (Y/N)
19	Adult nutritional RTF, high-fat	GOS 3	6	0.226	154	6.2	17.1	N
		GOS 2	6	0.586	127	4.3	12.0	N
		GOS 1	6	0.913	109	8.2	22.8	N
		GOS 2	6	3.01	111	5.0	13.9	N
24	Infant formula powder with partially hydrolyzed protein	GOS 1	6	0.205	8.50	375	1039	N
		GOS 3	6	0.655	102	20.7	57.5	N
		GOS 2	6	1.040	107	8.9	24.6	N
		GOS 3	6	3.00	109	1.5	4.0	Y
12	Child formula powder	GOS 2	6	0.203	157	21.3	59.1	N
		GOS 1	6	0.571	97.2	14.6	40.5	N
		GOS 3	6	1.150	126	31.3	86.6	N
		GOS 1	6	3.01	98.2	7.7	21.3	N
13	Infant elemental powder	GOS 3	6	0.231	137	21.5	59.7	N
		GOS 2	6	0.570	114	13.7	37.9	N
		GOS 1	6	1.01	95.9	28.5	21.7	N

**Table 10.** Precision of the method for the determination of GOS having a DP ≥ 3

No.	Sample description	*n*	GOS concn., g/100 g	GOS DP ≥ 3, g/100 g	RSD_r_, %	RSD_iR_, %
5	Infant formula RTF, milk-based	6 × 2	0.216	0.116	2.72	13.0
7	Infant formula powder partially hydrolyzed milk-based	6 × 2	0.357	0.234	0.78	7.68
14	Infant formula powder, FOS/GOS-based	6 × 2	0.333	0.229	3.28	8.74
15	Infant formula powder, milk-based	6 × 2	0.300	0.201	5.63	9.58
17	Infant formula powder, RTF milk-based	6 × 2	0.211	0.120	3.22	12.4
20	Infant formula powder with GOS	6 × 2	0.436	0.288	3.04	7.08
21	Infant formula powder with GOS	6 × 2	0.277	0.192	1.52	9.93
22	Infant formula powder with GOS/FOS	6 × 2	0.769	0.517	2.50	6.00
23	Adult nutritional RTF with GOS	6 × 2	0.664	0.435	8.80	12.1
